# Impact of substerilizing dose on histological changes in gonads and ovaries of *Ephestia cautella* (Lepidoptera: Pyralidae) by gamma radiation

**DOI:** 10.1038/s41598-022-17309-6

**Published:** 2022-08-02

**Authors:** Ali Hamza, Nagwan Zahran, Sameh Sawires

**Affiliations:** Department of Natural Products Research, National Center for Radiation Research and Technology (NCRRT), Egyptian Atomic Energy Authority (EAEA), Cairo, Egypt

**Keywords:** Structural biology, Zoology

## Abstract

To investigate the effect of Gamma radiation on the reproductive systems of emerged female and male of *Ephestia cautella* (Walker) moths, mature pupae of both sexes were irradiated with doses of 50, 100 and 150 Gy. Histological study of the treated individuals showed in females that the ovaries appear sever damage in the follicular epithelium at all doses, which become thinness and separated from developing oocytes, moreover, some of the nurse cells were rupture. In males which treated with 150 Gy, it was noticed retardation in the stages of spermatogenesis and few numbers of sperm bundles and their dispersion in the testicular follicles’ adults, on the other hand, the doses of 50 and 100 Gy showed little or moderate effects on the structure of the testis contents.

## Introduction

The almond moths, *Ephestia cautella* represents one of the most important insect pests in Egypt. It is one of the destructive pests of dried fruits, nuts, flour, grains and garlic. This insect has nocturnal activity in nature. Although it is usually active at dusk and early morning when there are fluctuations in temperature and relative humidity, it can fly even during the day. The larvae make several damage, it mostly feeds on germ portion and produces a lot of silken webs which join the grains together and contaminate food items with faecal matter. Webbing of grains produces lumps resulting into clogging in mills. In heavy infestation, the surface of the entire stock can be covered by silken webs made by the wandering larvae.

The problems of the widespread use of insecticides to control pests of stored products; led to the establishment of much safer or effective means to control insects. Among these approaches is ionizing radiation to induce insect sterilization. Although the sterile insect technique has often been associated with an eradication strategy, major advances in rearing efficiency, and improved handling and release methods have made the use of sterile insects economically feasible for insect pest suppression, prevention or containment^1^**.** The advantages of irradiation processing include no undesirable residues in the foods treated, no resistance developed by pest insects and few significant changes in the physic-chemical properties or the nutritive value of the treated products^2,3^**.**

The use of gamma radiation for controlling pests in grain and other stored commodities is one of the promising alternatives in this respect since different doses of gamma radiation reduced population size and mating ability. However insect irradiation could cause somatic damage that would interfere with biological aspects of the insects^4^**.** The degree of damage**,** due to irradiation in the reproductive system of pests depends on the age and irradiation dose. Histological studies on irradiated and nonirradiated ovaries and testes were carried out by same workers such as EL_Halafawy^5^ on *Spodoptera littoralis*, Ibrahim et al.^6^ on *Agrotis ipsilon,* Hazaa et al.^7^ on *Spodoptera littoralis* and Abdel Baki^8^ on *Plodia interpunctella*.

The present investigation is under taken to study the histological effects of substerilizing dose of gamma radiation on the ovaries and testes of emerged adults moth of *Ephestia cautella*.

## Materials and methods

### Rearing technique

Standard laboratory culture of the Almond moth, *Ephestia cautella* has been maintained at the Department of Natural Products, National Center for Radiation Research and Technology (NCRRT), Atomic Energy Authority (AEA), Cairo, Egypt. The Almond moth larvae were kept in a medium consisting of (65% crushed wheat, 10% sugar, 15% glycerin and 10% Brewer's yeast to 1 kg of media), at 26 ± 1 °C and 70 ± 5R.H.

### Irradiation technique

Irradiation was achieved using Gamma Cell ^60^Co source Irradiation Unit, at the National Center for Radiation Research and Technology (NCRRT), (EAEA), Cairo, Egypt. The dose rate was 1.05 KGy/hour at the time of experiment. Irradiated *Ephestia cautella* male and female (7 days old pupae) were placed separately in gelatin capsules until adult emergence.

### Histopathological studies

Reproductive system for both male and female moths which were irradiated and unirradiated with gamma radiation were dissected in Ringer physiological solution and after the gonads were freed from any fat body fixation were conducted in FAA (formalin acetic alcohol) for 2 days. Dehydration was through ascending graded series of ethanol for 12 h. and finally through absolute ethanol for 10 h. Clearing of samples was conducted firstly in a mixture of absolute ethanol and xylene (1:1) for 2 days and then in pure xylene overnight in an oven set at 65 °C and specimens embedded in pure melted paraffin wax (mp 58–60 °C) over night. Samples were blocked in the suitable orientation. Serial longitudinal and cross sections were cut at 8 u thicknesses using a manual microtone. Ribbons were mounted and adhered with Hayers media (egg albumin and glycerin (1: 1). The sections were stained with Ehrlich shaematoxyline, and counter skined in eosin technique.

## Results and discussion

### Histological structure of female reproductive system

Female reproductive system in *Ephestia cautella* as in most other lepidopterous insects is composed of two identical ovaries. Each ovary consists of four ovarioles of polytrophic type. Each ovary has a lateral oviduct. The two oviducts connect together to form the common oviducts^9^**.**

As for the polytrophic type ovariole, each ovariole is surrounded by a thin epithelial membrane (the outer sheath). Its apical part is called the terminal filament. The terminal filaments of the four ovarioles of each ovary are united together in a main filament. The two main filaments of both ovaries are united, together, to form a suspensory filament.

**Figure 1:**
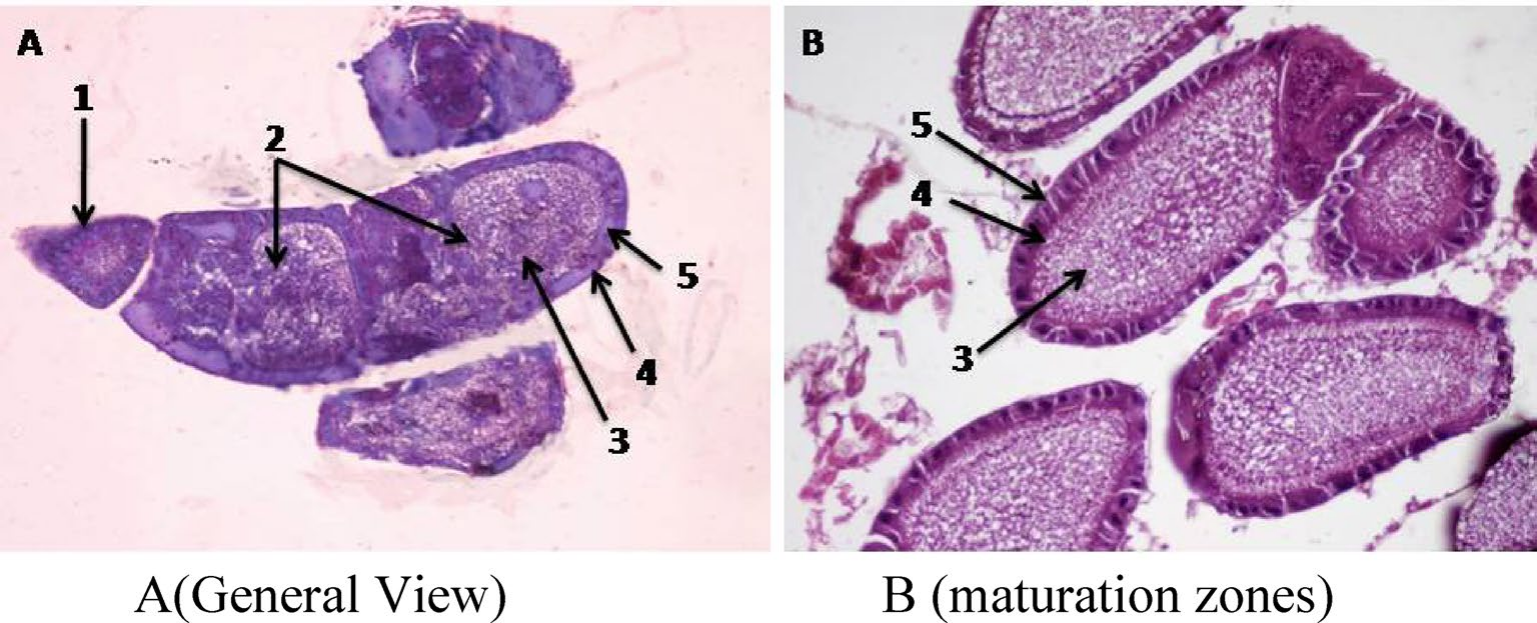
**(A,B)**. Cross section in the ovariole of normal (unirradiated) female *Ephestia cautella* (×400). 1, Germarium zone; 2, oocytes; 3, nurse cells; 4, epithelial cells; 5, follicular cells.

The upper part of each ovariole is called the germarium zone, which containing the promordial germ cells (oogonia, trophocytes and prefollicular cells), which later in vitelarium zone become differentiated until being mature to become eggs. Each oocyte is surrounded by follicular epithelium and has nurse cells arranged on its top and enclosed in its follicle. The oocytes and the nurse cells differ in size according to the stage of development when the oocytes become into a mature egg the nurse cells degenerate (Fig. 1A,B).

**Figure 2:**
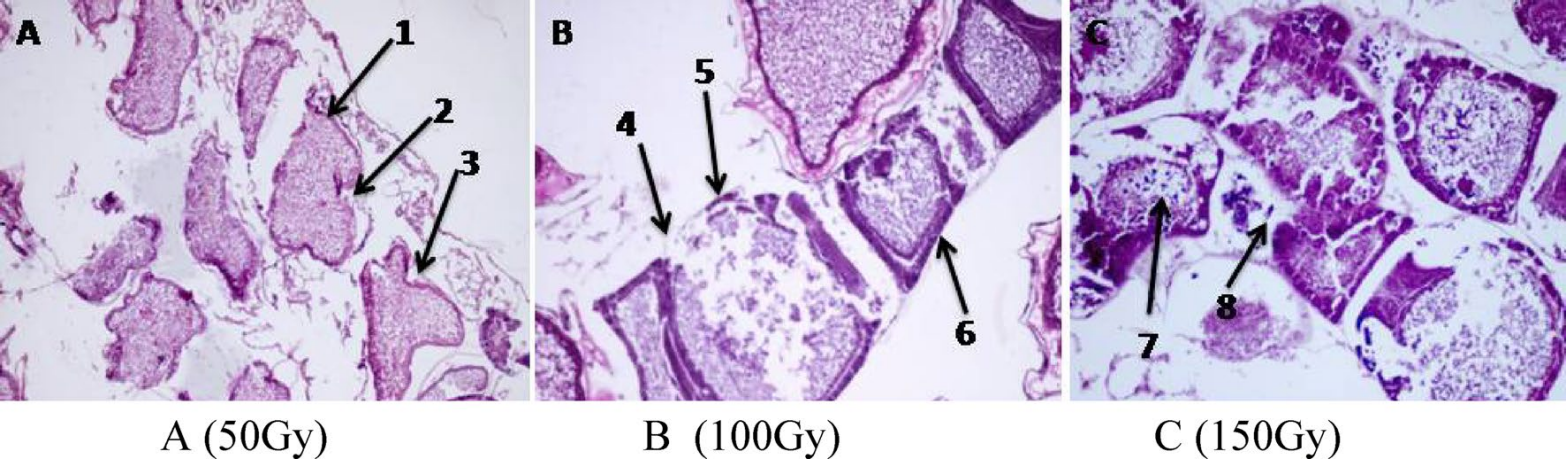
**(A–C)** Longitudinal sections in ovarioles of female emerging from pupae irradiated with gamma rays. 1, Rectangular oocyte; 2, absence of nurse cells; 3, irregular oocyte; 4, rupture of the epithelial cells; 5, absence of the epithelial cells; 6, shrinking oocyte; 7, deterioration; 8, separation.

A female emerging from pupae irradiated with 50, 100 and 150 Gy show various degrees of damage in the ovariole either in membranes or in the formation of the oocytes (Fig.2A–C). The effects on the membranes included the following absence of the epithelial follicular cells; rupture of the follicular cells and separation between the developing oocytes and the follicular epithelium. Alternation in the developing oocytes could be summarized as follows: a shrinkage in the oocyte contents leading to lose their oval shape causing a clear space around it; partial absence of the nurse cells and partial deterioration in the oocyte’s contents.

At a dose of 50 Gy the ovarioles appears degenerated with irregular separated oocytes and destruction of some follicular epithelial cell lining (Fig. 2A), also female exposed to 100 Gy, microscopic examination showed rupture of some ovarioles outer sheath and degeneration of oocytes with wide space between them (Fig. 2B), moreover degeneration of oocytes with separation of its epithelial cell lining at a dose of 150 Gy was observed (Fig. 2C).

**Figure 3:**
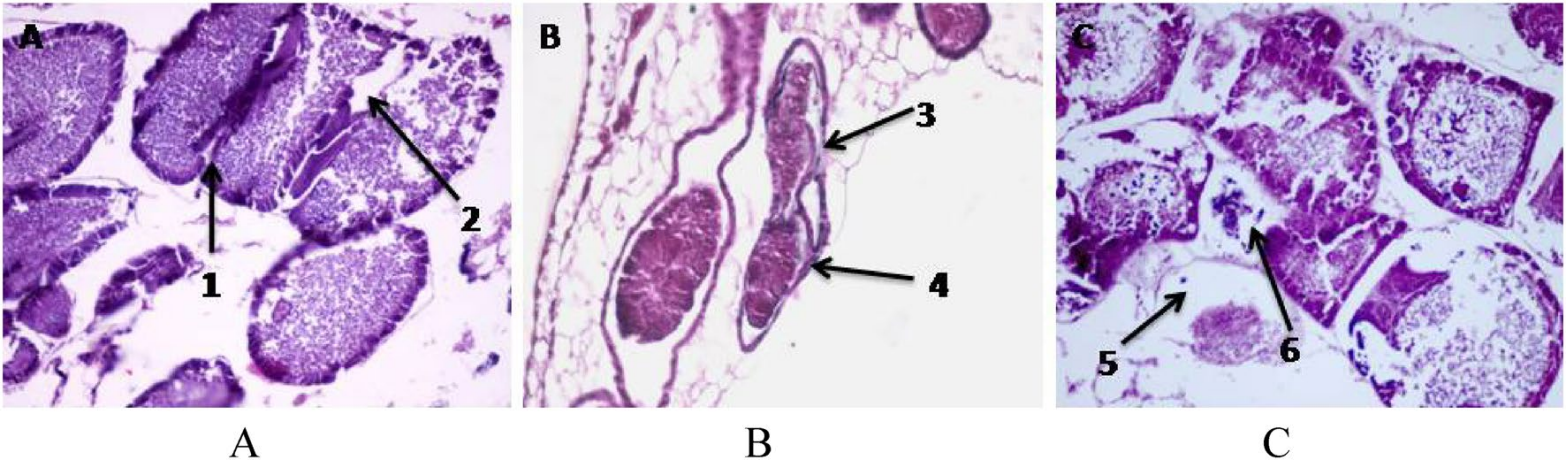
**(A–C)** Longitudinal sections in ovarioles of adult *Ephestia cautella* emerged from full grown pupae irradiated with (**A**) (50 Gy), (**B**) (100 Gy) and (**C**) (150 Gy) ×400. 1, Deterioration; 2, separation; 3, shrinking oocyte; 4, abnormal oocyte; 5, absence oocyte, 6, vacuole.

The deleterious effects of radiation on the ovaries of *Ephestia cautella* increased with the increase of the radiation dose and they were more pronounced in female moths irradiated as pupae at the dose of 150 Gy. Histological observations in the ovaries as illustrated in Fig. 3A show degeneration and separation in the oocytes which became rectangular in shape, through the ovaries of female moths previously irradiated as full grown pupae at 50 Gy. Figure 3B shows that the irradiation at 100 Gy caused malformed oocytes and appeared clumped, semi-absorbed or degenerated leaving vacuoles everywhere. The dose of 150 Gy caused severe ovarian histological damages such as complete rupture of the ovariole outer sheath, the oocytes appeared abnormal and some of them looked irregular in shape with a complete absence of the nurse cells Fig. 3C.

### Histological structure of the male reproductive system

The male reproductive system of newly emerged *Ephestia cautella* is composed of two testes in a scortum which appears as a spherical white yellowish structure covered with a thin wall. Each testis consists of four testicular follicles (chambers) that open in the vasa differentia. The two vasa differentia are united together to from a common ejaculatory duct which terminates at the base of aedeagus.

**Figure 4:**
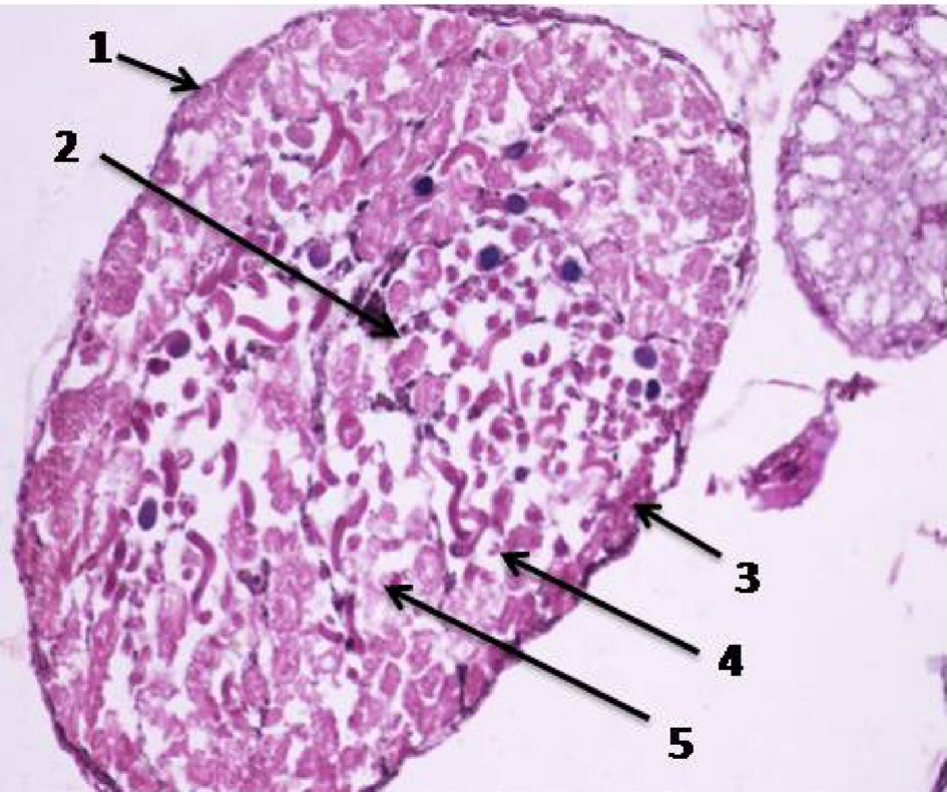
Cross section in the testis of unirradiated male *Ephestia cautella* (×400). 1, Testicular wall; 2, spermatogonia; 3, spermatids; 4, primary spermatocyte; 5, secondary spermatocyte.

Examination of the transverse section of unirradiated *E. cautella* male showed that there are various stages of sperm development within the adult testis. Each testicular follicle consists of a germarium and the growth zone in which the spermatogonial cells (primary and secondary spermatocytes) occupy the peripheral region of the testis. The maturation zone the part were the spermatocytes transform by two meiotic divisions to spermatids that develop to spermatozoa in shape of bundles in the central area (Fig. 4).

**Figure 5:**
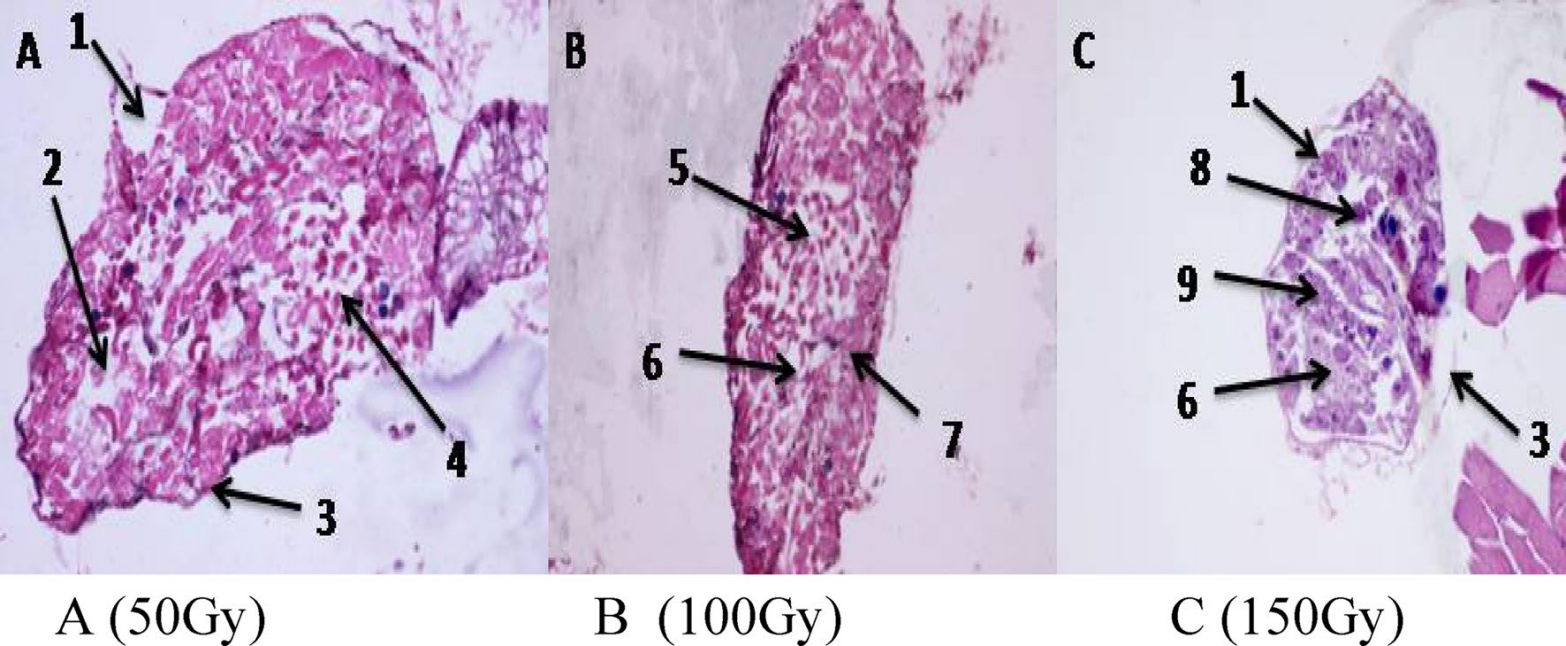
Cross section of testis tubules of irradiated male with (**A**) (50 Gy), (**B**) (100 Gy) and (**C**) (150 Gy). 1, Thin testicular wall; 2, absent spermatogonia; 3, irregular wall and ruptured testicular wall; 4, broken interfollicular partitions; 5, individual sperms; 6, absorbed spermatids; 7, broken sperm bundle and retardation in spermatogenesis; 8, dark masses; 9, clusters of spermatocytes.

Male moths emerging from irradiated pupae at doses of 50, 100 and 150 Gy showed a clear reduction in the size of testis which seemed irregular in shape as compared to the unirradiated males. The cross sections through the testis of moths showed various degrees of damage according to dose. The testicular wall became thinner and sometimes looked ruptured. Spermatogonia looked based on irregular surface. A clear separation or detachment of spermatogonia with an absence of some cells was observed. The interfollicular partition appeared broken. Some spermatocytes appeared in clusters and cannot be distinguished. Degeneration appeared all over the testis as dark colored masses. Sperm bundles were damaged or completely absent in many areas as large vacuoles appeared. Appearance of vacuoles in the stages of spermatogensis indicated an interruption of normal cell development in comparison to control (Fig. 5A–C).

Histological section of irradiated female ovariole showed different injury compared with that of the unirradiated female ovarioles. A dose of 150 Gy indused severe damages represented by thinness in the follicular epithelium and separation from developing oocytes when compared to those damages induced by 50 and 100 Gy. Moreover, the nurse cells sometimes, disappeared as compared with unirradiated female. The resulting malformation in the female reproductive organs and tissues are in harmony with those obtained by Hassaballa et al*.*^10^ on *Ephestia kuehniella* and Boshra & El-Naggar^11^ on *Plodia interpunctella*. The reduction of oocytes in ovaries, egg production and oocytes remaining in the ovaries were due to egg absorption. Similar results were obtained in previous publications by Mikhaiel^12^, Abd-Elwahed^13^, Hafez and Hamed^14^.

AbdEl-meguid and Haiba^15^ found that the ultrastructural adult females of *phthorimaea operculella* ovaries in irradiated insects that were exposed to substerilizing and sterilizing doses showed extreme deterioration in the ovarian development. In addition, some researchers like Abdel Baki and Al Khalaf^8^ investigated that irradiation of female pupae reduced the number of developing oocytes and fewer eggs were laid by *Plodia interpunctella.* This effect was attributed to the reduction in the length of ovariole and reabsorption of oocyte as compared to its occurrence in untreated moths.

The photographed effects included not only a damage of testis structure but also loss of sperm bundles, degeneration and malformations in the resulting sperm represent shrinkage and clumping. Ibrahim et al*.*^6^ irradiated males of *Ephestia cautella* with 50, 100 and 150 Gy of gamma radiation. Histological observation on the testes of adults showed different degrees of damage including shrinkage of testes contents, vacillations and disturbance in spermatogenesis. The damage increased as the dose given to males was increased. Most of testis contents became a mass, no longer distinguishable and mature sperm bandies appeared loose or broken.

A previous study by Mansour^16^ examined the effects of gamma irradiation on fertility and reproductive behavior of codling moth, *Cydia pomonella*. Results showed that egg production and hatching decreased with increasing the radiation dose, and females were more sensitive to radiation treatment than males. A dose of 150 Gy caused 100% sterility in females and significantly reduced fecundity, and a dose of 350 Gy caused 100% sterility in males. The resulting defects in testes are similar to those observed and photographed by Ibrahim et al*.*^6^ on *Agrotis ipsilon.*

An earlier publication by Alm EL-Din^17^ studied the histological effects on the ovaries and testes of F_1_ females and males of *Spodoptera littoralis* resulted from parental male irradiated as full-grown pupae at doses of 25, 50, 75 and 100 Gy. The damage occurred was positively correlated with the dose given to P males. The damage was observed in the ovarioles including thinness in the follicular eipthelium and their separation from developing oocyte; clumped ooplasm, vacuolization of yolk, reduction, deformation in the nurse cells and sometimes their absence. Sawires^18^ exposed full grown male and female pupae of Mediterranean flour moth, *E. kuehniella* to doses of gamma rays ranged from 50 to 450 Gy. He found that males were more radio- resistant than females.

Finally, it could be commented that the emerged high doses of gamma radiation showed increasing grades of deformities in morphology for both male and female moths. These deformities inmorphology led to reduced mating competitiveness of both treated males and females and also reduced fertility.

## Conclusion

The results presented in the current study lead to the conclusion that subserializing dose of radiation induces damage to the ovarian and testes structure and inhibition of egg development. Thus no fertile eggs can be produced and the reduction in egg hatch may be explained by dominant lethal mutation, carried in the sperm nuclei and or by decrease in sperm mortality. It can be concluded that the reproductive system in *E. cautella* is damaged by irradiation and this effect increases as the dose increases.

## Data Availability

All data generated or analyzed during this study are included in this published article.

## References

[1] Marc JB, Alan SR (2011). Ionizing radiation and area-wide management of insect pests to promote sustainable agriculture. A review. Agron. Sustain. Dev..

[2] Ahmed M, Molins R (2001). Disinfestation of stored grain, pulses, dried fruits and nuts, and other dried foods. Food Irradiation Principles and Applications.

[3] Boshra SA, Mikhaiel AA (2006). Effect of gamma irradiation on pupal stage of *Ephestia calidella* (Guenée). J. Stored Prod. Res..

[4] Ahmed, Z. A. Combined effect of gamma radiation and some insecticides on certain stored product insects. *Ph. D Thesis*. Faculty of Science, Cairo University (1992).

[5] EL_Halafawy, N. A. Effect of certain radio active compounds on the reproduction and reproductive system of the cotton leaf worm *Spodoptera littoralis*. *M.Sc. Thesis*. Faculty of Science, Ain Shams University (1983).

[6] Ibrahim SM, El-Naggar SEM, El-Shall SSA (1999). Inheritance of radiation induced partial sterility among F1 larval and adult males of the cutworm, *Agrotis ipsilon* (Hufn), (histological studies). Arab. J. Nucl. Sci. Appl..

[7] Hazaa MAM, Alm El-Din MMS, El-Akhdar EAH (2009). The histological and histochemical changes in the gonads of the cotton leaf worm *Spodoptera littoralis* (boisd.). Isot. Radiat. Technol..

[8] Abdel Baki SM, Al Khalaf AA (2019). The effect of gamma irradiation on the ovaries and testes of *Plodia interpunctella* (Phycitidae: Lepidoptera). Adv. Agric. Eng..

[9] Jin, X. U. Reproductive behaviour of *Ephestia Kuehniella* (Zeller). *PhD. Thesis*. Massey University, Palmerston North (2010).

[10] Hassaballa ZA, Rizk MMA, Ibrahim SM (1987). Histological changes in the ovaries of *Ephestia kuehniella* females irradiated as fully-grown larvae by gamma radiation. Assiut J. Agric. Sci..

[11] Boshra SA, El-Naggar SEM (1994). Effects of gamma radiation on reproductive biology and histology of sex pheromone glands and ovaries of female *PIodia interpunctella *(Hubner). Bull. Entomol. Soc. Egypt. Ser..

[12] Mikhaiel, A. A. The effect of gamma irradiation on Oases date moth, *Ephestia caldiella *(Guen). *PhD Thesis*. Faculty of Science, Cairo University (2003).

[13] Abd-Elwahed, S. M. Biological and histological studies on the effects of gamma irradiation entomopathogenic nematodes and some plant extracts on potato tuber Moth *phthorimaea operculella* (Zeiler). *PhD Thesis*. Faculty Girls Ain Shams University (2004).

[14] Hafez, S. E. & Hamed, R. K. Effect of permethrin and diflubenzuron on the line structure of the ovary of *Spodoptera littoralis* (Boisd) (Lepidoptera: Noctuidae). *Egypt. J. Zool.* (2004).

[15] AbdEl-meguid A, Haiba IM (1996). Effect of gamma irradiation on the ovaries of the potato moth, *Phthorimaea operculella*zeller (Lepidoptera: Gelechiidae), An ultrastructural study. Arab J. Nucl. Sci. Appl..

[16] Mansour M (2002). Gamma radiation as a quarantine treatment for apples infested by codling moth (Lep. Tortricidae). J. Appl. Entomol..

[17] Alm EL-Din, M. M. S. Studies on inherited sterility induced in the progeny of gamma irradiated cotton leaf worm*, Spodoptera littoralis.**M.Sc. Thesis*. Faculty of Agriculture, A1-Azhar University (1993).

[18] Sawires, S. G. N. Biological and Biochemical Effects of Gamma Irradiation on the Mediterranean Flour Moth, *Ephestia kuehniella* (Zell.). *M.Sc. Thesis*. Faculty of Agriculture, Ain Shams University (2005).

